# Exploring the Therapeutic Composition and Mechanism of Jiang-Suan-Chu-Bi Recipe on Gouty Arthritis Using an Integrated Approach Based on Chemical Profile, Network Pharmacology and Experimental Support Using Molecular Cell Biology

**DOI:** 10.3389/fphar.2019.01626

**Published:** 2020-01-31

**Authors:** Nan Xiao, Jialin Qu, Shiyong He, Peng Huang, Yanling Qiao, Guangxing Li, Taowen Pan, Hua Sui, Lin Zhang

**Affiliations:** ^1^ Institute of Integrative Medicine, Dalian Medical University, Dalian, China; ^2^ Clinical Laboratory of Integrative Medicine, The First Affiliated Hospital of Dalian Medical University, Dalian, China

**Keywords:** Jiang-Suan-Chu-Bi recipe (JSCBR), gouty arthritis, chemical profile, network pharmacology, NOD-like receptor signaling pathway

## Abstract

**Background:**

Gouty arthritis is a common metabolic disease caused by long-term purine metabolic disorder and elevated serum uric acid. Jiang-Suan-Chu-Bi recipe (JSCBR), a traditional Chinese herbal formula prescribed according to utilization frequency and cluster analysis, has been clinically validated remedy for gouty arthritis. However, its therapeutic composition and mechanism remains unclear.

**Methods:**

In the present study, a simple, rapid, and sensitive ultraperformance liquid chromatography coupled with quadrupole time-of-flight mass spectrometry (UHPLC-QTOF-MS)-based chemical profiling was firstly established for comprehensively identifying the major constituents in JSCBR. A phytochemistry-based network pharmacology analysis was further performed to explore the potential therapeutic targets and pathways involved in JSCBR bioactivity. Finally, THP-1 cell model was used to verify the prediction results of network pharmacology by western blot analysis.

**Results:**

A total of 139 compounds containing phenolic acids, flavonoids, triterpenoid saponins, alkaloids, amino acids, fatty acids, anthraquinones, terpenes, coumarins, and other miscellaneous compounds were identified, respectively. 175 disease genes, 51 potential target nodes, 80 compounds, and 11 related pathways based on network pharmacology analysis were achieved. Among these pathways and genes, NOD-like receptor signaling pathway may play an important role in the curative effect of JSCBR on gouty arthritis by regulation of NRLP3/ASC/CASP1/IL1B. The results of cellular and molecular experiments showed that JSCBR can effectively reduce the protein expression of ASC, caspase-1, IL-1β, and NRLP3 in monosodium urate-induced THP-1 cells, which indicated that JSCBR mediated inflammation in gouty arthritis by inhibiting the activation of NOD-like receptor signaling pathway.

**Conclusion:**

Thus, the integrated approaches adopted in the present study could contribute to simplifying the complex system and providing directions for further research of JSCBR.

## Introduction

Gouty arthritis is a metabolic disease caused by the deposition of monosodium urate crystals in joints and soft tissues (Pascart et al., 2018) and closely associated with chronic hyperuricemia, which seriously affects quality of life of patients due to severe pain (Nielsen et al., 2018). Across the globe, the age of onset is getting younger and younger (Zhou et al., 2014). It is estimated that the number of gout patients in China will exceed 100 million in 2020. Now, gout has become the second largest metabolic disease in China (Wilson and Saseen, 2016).

At present, western medicines including colchicine, allopurinol, benzbromarone, and febuxostat have been used as the conventional therapy method for gouty arthritis. Although their short-term effect on inhibiting uric acid production, promoting uric acid excretion, or analgesic effect is optimistic, the side effects such as skin mucosal injury, kidney and liver injury, digestive tract injury, as well as some potential neurotoxicity and muscular toxicity caused by long-term use could not be ignored. Meanwhile, as the main form of traditional Chinese medicine (TCM) that based on the compatibility theory, traditional Chinese herbal formula has been applied for the treatment of gouty arthritis for thousands of years in view of remarkable therapeutic effect and little adverse reactions (Zhou et al., 2014).

Jiang-Suan-Chu-Bi recipe (JSCBR) is a formula prescribed according to utilization frequency and cluster analysis by retrieving gout-related data obtained from multiple databases (Xiao et al., 2019), which has been clinically validated remedy for gouty arthritis under the guidance of TCM doctors. It consists of 12 herbs, namely, Smilax glabra Roxb. (tu-fu-ling in Chinese, TFL, batch No. 20160925), Paeonia lactiflora Pall (chi-shao in Chinese, CS, batch No. 20160814), Reynoutria japonica Houtt. (hu-zhang in Chinese, HZ, batch No. 20160728), Cremastra appendiculata (D.Don) Makino (shan-ci-gu in Chinese, SCG, batch No. 20160723), Rheum officinale Baill. (da-huang in Chinese, DH, batch No. 20160719), Clematis chinensis Osbeck (wei-ling-xian in Chinese, WLX, batch No. 20160915), Angelica pubescens Maxim. (du-huo in Chinese, Dh, batch No. 20160822), Phellodendron chinense C.K.Schneid. (huang-bai in Chinese, HB, batch No. 20160918), Atractylodes lancea (Thunb.) DC. (cang-zhu in Chinese, CZ, batch No. 20160814), Glycyrrhiza glabra L. (gan-cao in Chinese, GC, batch No. 20160816), Codonopsis pilosula (Franch.) Nannf. (dang-shen in Chinese, DS, batch No. 20160723), and Angelica sinensis (Oliv.) Diels (dang-gui in Chinese, DG, batch No. 20160801). Although its prescription is scientific and the efficacy is definite. To date, no reports on the chemical composition and functional mechanisms of JSCBR contribute to its bioactivity has been published, which restrict its further application and development.

**Figure 1:**
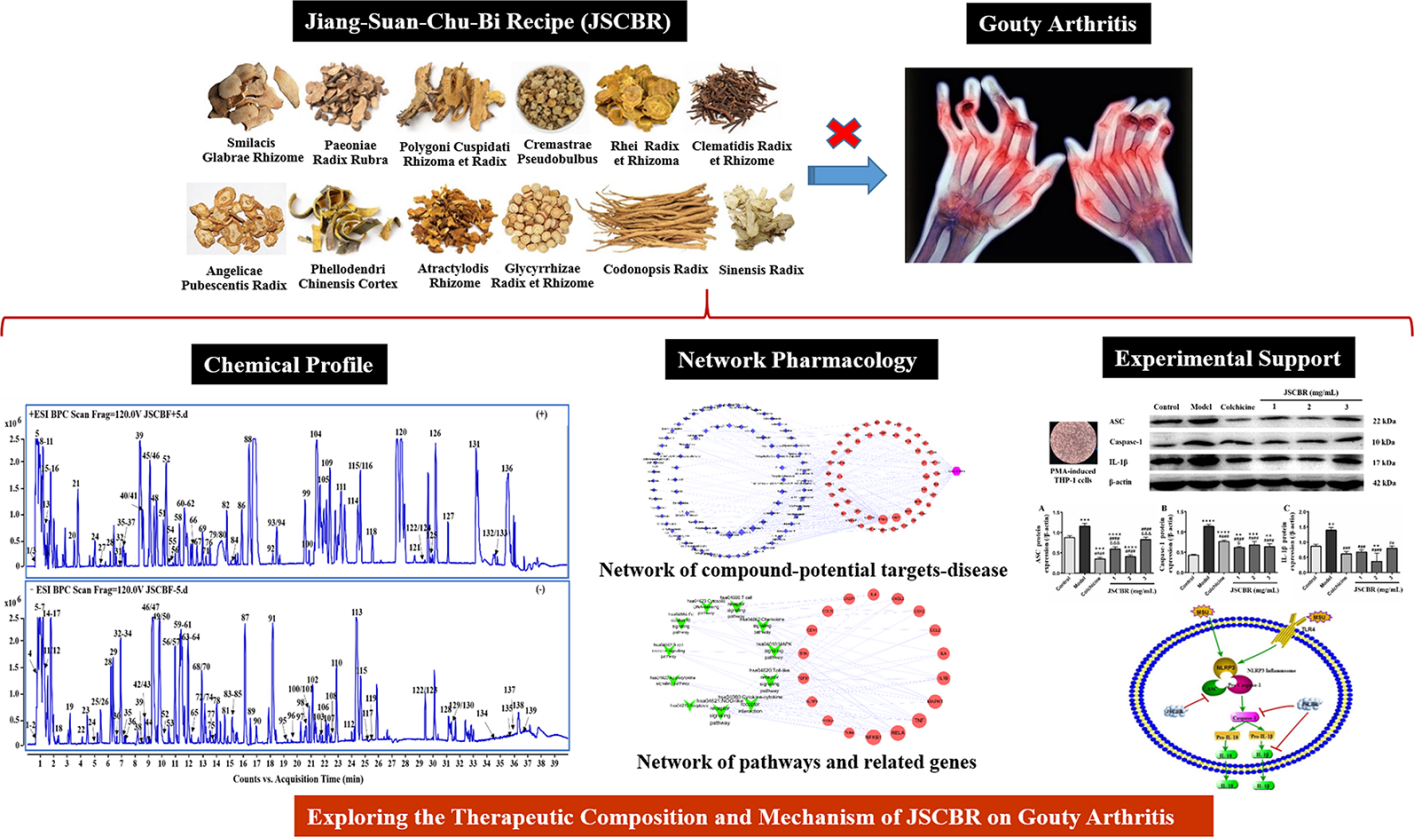
The schematic diagram of the present study.

In the present study, a systematic dissection of JSCBR was employed by integrating chemical profile, network pharmacology, and experimental support using molecular cell biology. The schematic diagram of the present study was depicted in **Figure 1**.

## Materials and Methods

### Chemicals, Reagents, and Materials

UHPLC-MS grade acetonitrile and methanol were supplied by Merck Company Inc., (Darmstadt, Germany). MS grade formic acid was purchased from Fisher Scientific Company (Inc., Fairlawn, NJ). Ultrapure water (18.2 MΩ) was prepared with a Milli-Q water purification system (Millipore, Milford, MA, USA). All other reagents were of analytical grade and purchased from Tianjin Concord Technology Co. Ltd. (Tianjin, China).

The reference compounds ferulic acid (**56**), polydatin (**57**), paeoniflorin (**79**), atractylodin (**81**), liquiritin (**84**), aloe-emodin (**94**), rhein (**115**), glycyrrhizic acid (**119**), osthole (**126**), columbianadin (**127**), and oleanolic acid (**134**) were purchased from Chengdu Pufei De Biotech (Chengdu, Sichuan, China). The reference compounds astilbin (**60**), chysophanol (**93**), physcion (**100**), and emodin (**122**) were purchased from Baoji Herbest Biotech (Baoji, Shanxi, China). The purity of each reference standard was determined to be over 98% by UHPLC analysis. All the 12 herbs of JSCBR were purchased from Beijing Tong-Ren-Tang Technologies Co., Ltd. (Taiyuan, Shanxi Province, China), and authenticated by Professor Yunpeng Diao (College of Pharmacy, Dalian Medical University). Voucher specimens were deposited at the authors’ laboratory.

THP-1 cell was purchased from Yipu Biological Technology Co., Ltd. (Wuhan, Hubei Province, China). RPMI 1640 cell culture mediums and pencillin-streptomycin were purchased from Gibco BRL, Invitrogen Corporation (Grand Island, NY). Fetal bovine serum was purchased from Zhejiang Tianhang Biotechnology Co., Ltd. (Hangzhou, Zhejiang Province, China). Cell Counting Kit -8 (CCK-8) was purchased from Yiyuan Biotechnology Co., Ltd. (Guangzhou, Guangdong Province, China). Phorbol-12-myristate-13-acetate (PMA) was purchased from Abcam Ltd., (Cambridge, UK). Monosodium urate (MSU) was purchased from Sigma-Aldrich (St Louis, MO, USA). Colchicine was supplied by Shengshi Kangpu Chemical Technology Research Institute (Beijing, China). Antibodies against caspase-1, β-actin, apoptosis-associated speck-like protein (ASC), IL-1β, and antirabbit IgG-HRP were purchased from Bioss (Beijing, China). The BCA Protein Quantification Kit was purchased from KeyGen Biotech Co., Ltd. (Nanjing, Jiangsu Province, China). RIPA lysis buffer and PMSF protease inhibitor were purchased from Beyotime Institute of Technology (Shanghai, China). ECL-plus chemiluminescence reagent was purchased from Bio-rad (Richmond, CA, USA).

### Preparation of JSCBR Extract

The usage of each crude herb were accurately weighed as follows: Smilacis Glabrae Rhizome (30 g), Paeoniae Radix Rubra (10 g), Polygoni Cuspidati Rhizoma et Radix (15 g), Cremastrae Pseudobulbus (8 g), Rhei Radix et Rhizoma (10 g), Clematidis Radix et Rhizome (15 g), Angelicae Pubescentis Radix (10 g), Phellodendri Chinensis Cortex (10 g), Atractylodis Rhizome (10 g), Glycyrrhizae Radix et Rhizome (10 g), Codonopsis Radix (15 g), and Angelicae Sinensis Radix (10 g). They were mixed and prepared by the decocting method as described below: a total of 153 g mixture was immersed in sevenfold mass of water for 2 h, heated and refluxed for 2 h and then filtered with six-layer absorbent gauze. A fivefold mass of water was subsequently added to the residues and boiled for 2 h. After being filtered with six-layer absorbent gauze, the two filters were combined and concentrated to 150 ml (equal to 1 g crude herb/ml). The liquid was finally transformed into powder by vacuum freeze drying technology.

A 1.0 g of the freeze-dried powder was accurately weighted and extracted with 50 ml of methanol/water (1:1, v/v) for 30 min under ultrasound. The extract solution was centrifuged at 13,000 rpm for 10 min at 4°C and the supernatant was filtered through a 0.22 μm membrane filters. 1.0 μl of filtrate was injected to UHPLC-QTOF-MS for analysis.

### UHPLC-QTOF-MS Analysis

The chromatography and MS conditions were almost the same as those reported in literature (Huang et al., 2019). The only difference is the change of elution gradient, which was listed as follows: 0–25 min, 5%–45% B; 25–35 min, 45%–99% B; 35–38 min, 99%–5% B; 38–40 min, 5% B.

### Establishment of In-House Library for JSCBR and Generation of Empirical Molecular Formula

The in-house library that covers all previous reported compounds from the 12 formulated herbs was established in a Microsoft office excel table, which includes compound name, molecular formula, molecular weight, chemical structures, natural source, and related references (Chen et al., 2010; Liang et al., 2013; Ge et al., 2014; Ma et al., 2014; Bai et al., 2015; Guo et al., 2015; He et al., 2016; Sun et al., 2016; Liu et al., 2018; Wang et al., 2018; Xu et al., 2018). The empirical molecular formula that matched the criterion for ppm below 10 were deduced as potential candidates by the function “Find Compounds by Formula” of Agilent MassHunter qualitative analysis software. Only those compounds that had been compared with standards or characteristic fragment ions were finally selected as chemical composition of JSCBR.

### Network Pharmacology Analysis

#### Identification of Candidate Targets of Gouty Arthritis

Gouty arthritis related targets were acquired from seven existing resources: 1. TTD database (http://bidd.nus.edu.sg/BIDD-Databases/TTD/TTD.asp); 2. OMIM database (http://omim.org/); 3. PharmGKB database (https://www.pharmgkb.org/); 4. DrugBank database (http://www.drugbank.ca/, version 4.3); 5. GAD database (https://geneticassociationdb.nih.gov/); 6. DisGeNET database (http://www.disgenet.org/web/DisGeNET/menu); 7. GeneCards database (https://www.genecards.org/). We searched these databases and acquired 175 genes totally after removing duplicates. The detailed information is provided in **Supplementary Table S1**.

#### Identification of Compound Targets of JSCBR

After identifying the compounds contained in JSCBR by UHPLC-QTOF-MS, the CAS number and Canonical SMILES were collected by PubChem Compound (https://www.ncbi.nlm.nih.gov/pccompound/). In order to get as many targets as possible, two databases were employed to this study, including Swiss Target Prediction (http://www.swisstargetprediction.ch/) and Traditional Chinese Medicine Systems Pharmacology Database and Analysis Platform (TCMSP, http://lsp.nwu.edu.cn/tcmsp.php). Then, the gene information including name, gene ID, and organism was confirmed using UniProt (http://www.uniprot.org/). The final selected genes are supplied in **Supplementary Table S2**.

#### The Protein-Protein Interactions (PPIs) Network Analysis of Disease Targets

The protein-protein interactions (PPIs) network of gouty arthritis was constructed and analyzed by STRING database. Those PPIs with high confidence score (>0.95) should be selected for network construction and analysis to ensure the accuracy of the results.

### Network Construction and Analysis

All the networks can be performed by employing the network visualization software Cytoscape 3.2.1. Three networks were constructed as follows: (1) PPIs of gouty arthritis targets; (2) Compound-potential targets-disease network analysis; (3) Pathways-targets network analysis. In these network plots, nodes represent herbs and chemicals, and edges indicate interactions between different chemicals, targets, and pathways. The “degree” of a node was defined as the number of its connected edges.

### Enrichment Analysis

To clarify the signaling pathways and functions of potential target genes, Kyoto Encyclopedia of Genes and Genomes (KEGG) pathway enrichments analysis was performed based on Database for Annotation, Visualization and Integrated Discovery (DAVID, https://david.ncifcrf.gov/home.jsp, ver. 6.8) and STRING.

### Experimental Validation Using Molecular Cell Biology

#### Cell Culture

Human monocytic leukemia THP-1 cells were cultured in DMEM medium supplemented with 10% fetal bovine serum, penicillin (100 units/ml) and streptomycin (100 μg/ml). Cells were maintained in an incubator at 37˚C with 5% CO_2_ and saturated humidity and the culture medium was replaced with complete culture medium every 2 or 3 days.

#### CCK-8 Assay for Cell Viability

THP-1 cells were harvested during the logarithmic growth phase and seeded into a 96-well plate at a density of 1x10^6^ cells/well with a final volume of 100 μl. The cells were treated with PMA (100 nM) for 3 h to induce their differentiation into resting M0 macrophage. Fresh complete culture medium was added and cultured for 24 h after washing the cells with PBS solution. Differentiated THP-1 cells were then stimulated with MSU (100, 150, 200, 300, 400, and 500 μg/ml) with optimal concentration and treated with JSCBR extracts at final concentrations of 1, 2, 3, 4, and 5 mg/ml as well as colchicine (positive drug, 2 μg/ml) simultaneously at 37˚C for 24 h (Li et al., 2019). After treatment, 10 μl of CCK-8 was added to each well, and the plates were then incubated for additional 1.5 h. The absorbance at 450 nm was measured using 600 nm as a reference wavelength, which performed on a SpectraMax M2 reader (Molecular Devices, Silicon Valley, USA), and culture medium without cell was used as a blank. The experiments were performed in triplicate and repeated at least twice. The cell viability was calculated using the following formula:

$$Cell\;viability\;\left(\%\right)\;=\;\frac{A_{s}\;-\;A_{b}}{A_{c}\;-A_{b}}\;\times \;100\%$$

where A_s_, A_b_, and A_c_ represent the absorbance difference of experimental, blank, and control groups, respectively.

#### Western Blot Analysis

Total protein was extracted by incubation of cell pellet with RIPA lysis buffer and PMSF protease inhibitor. The protein concentration was determined by the BCA Protein Quantification Kit according to the instructions of manufacturer. The cell lysate containing 68 μg of protein was fractionated by 10% SDS-PAGE and then transferred to a poly (vinylidene difluoride) (PVDF) membrane. After blocking with 5% nonfat milk in Tris-buffered saline containing 0.1% Tween-20 at room temperature for 2 h, the membrane was incubated with primary antibodies at 4˚C overnight and horseradish peroxidase-conjugated secondary antibody for 2 h at room temperature, respectively. Protein bands were probed with an ECL Plus chemiluminescence reagent and exposed to a Tanon-5200 Imaging System (Tanon, Shanghai, China).

#### Statistical Analysis

Data are presented as means ± SD for three independent experiments. Statistical differences between two groups were analyzed using a Student’s t test by GraphPad Prism 5.0 (San Diego, CA, USA). A significant difference was considered as P < 0.05.

## Results

### Chemical Profile of JSCBR by UHPLC-QTOF-MS

**Figure 2:**
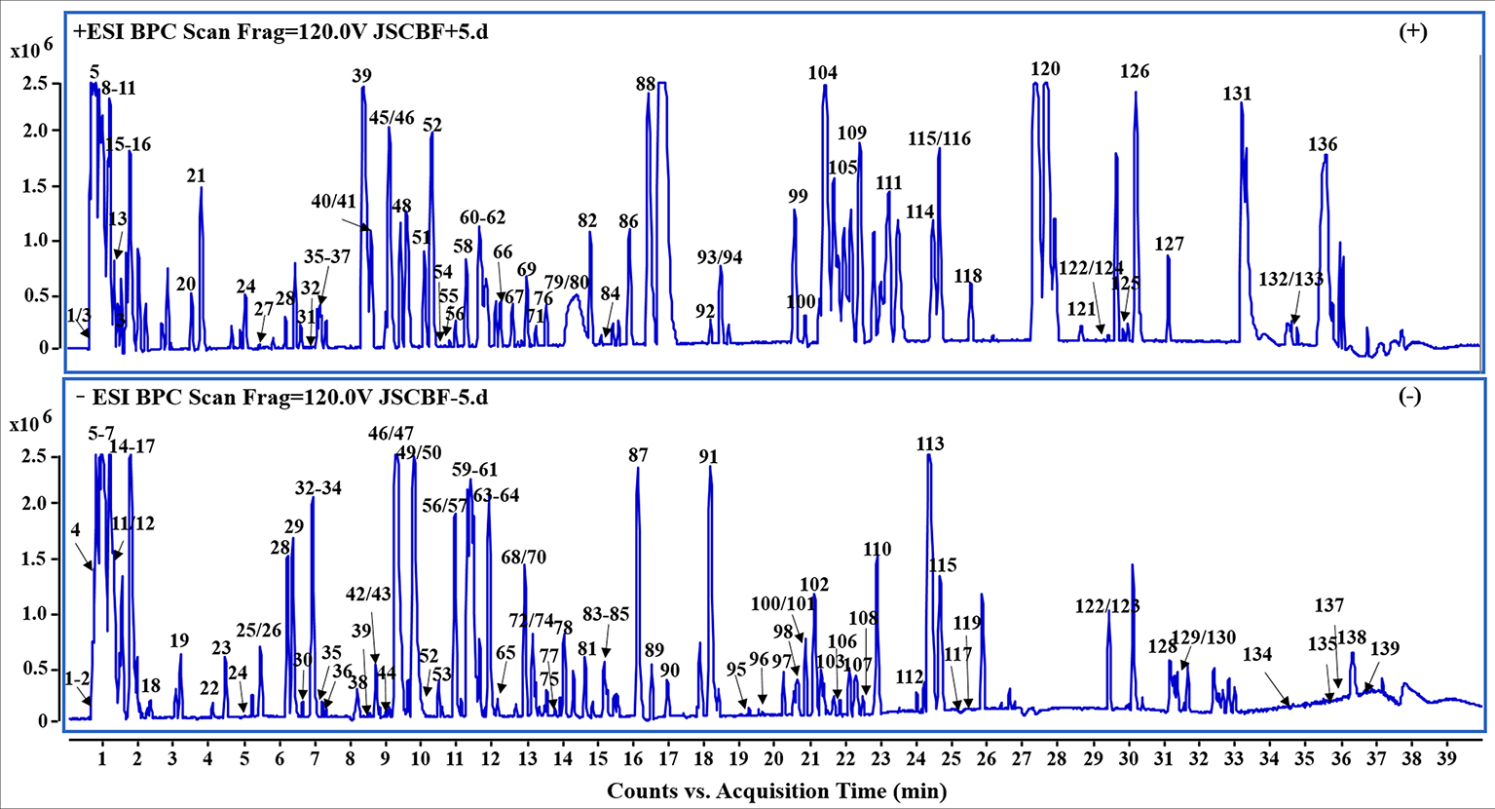
Representative base peak chromatogram (BPC) of Jiang-Suan-Chu-Bi recipe (JSCBR) in the positive and negative ions mode, respectively.

In the present study, a specific UHPLC-QTOF-MS protocol was performed to rapidly identify the compounds of JSCBR as many as possible after optimizing the LC and MS conditions systemically. As a result, a total of 139 compounds, including 39 phenolic acids, 19 flavonoids, 13 triterpenoid saponins, 11 alkaloids, 10 amino acids, 10 fatty acids, 8 anthraquinones, 8 terpenes, 6 coumarins, and 15 miscellaneous compounds were identified or tentatively characterized (**Figure 2**, **Table 1**).

**Table 1:** Characterization of the chemical constituents in Jiang-Suan-Chu-Bi recipe (JSCBR) by ultraperformance liquid chromatography coupled with quadrupole time-of-flight mass spectrometry (UHPLC-QTOF-MS).

Peak No	t_R_ (min)	Identification	Formula ion	Negative ion				Positive ion				Source *^a^*
				Quasi-molecular mass (Da)	Observed mass (Da)	Calculated molecular ion	ppm	Quasi- mass (Da)	Observed mass (Da)	Calculated	ppm	
1	0.766	Arginine	C_6_H_14_N_4_O_2_	[M-H] ^–^	173.1044	173.1044	-2.8	[M+H] ^+^	175.1191	175.1190	-0.6	GC/DS/DG
2	0.799	Fructose	C_6_H_12_O_6_	[M-H] ^–^	179.0566	179.0561	-2.8	—	—	—	—	DG
3	0.804	Pulegone	C_10_H_16_O	—	—	—	—	[M+Na] ^+^	175.1098	175.1093	-2.9	DS
4	0.816	Hypoxanthine	C_5_H_4_N_4_O	[M+CH_3_COO] ^–^	195.0517	195.0524	3.6	—	—	—	—	DG
5	0.853	Proline	C_5_H_9_NO_2_	[M-H] ^–^	114.0539	114.0561	19.3	[M+H] ^+^	116.0694	116.0706	10.3	GC/DG
6	0.866	Atractylenolide III sulfate	C_15_H_20_O_6_S	[M+CH_3_COO] ^–^	387.1152	387.1119	-8.5					DS
7	1.031	Malic acid	C_4_H_6_O_5_	[M-H] ^–^	133.0148	133.0142	-4.5	—	—	—	—	CZ
8	1.268	Pyroglutamic acid	C_5_H_7_NO_3_	—	—	—	—	[M+H] ^+^	130.0499	130.0499	0	GC/SCG
9	1.350	Tyrosine	C_9_H_11_NO_3_	—	—	—	—	[M+H] ^+^	182.0794	182.0812	9.9	GC/SCG/DG
10	1.384	Adenosine	C_10_H_13_N_5_O_4_	—	—	—	—	[M+H] ^+^	268.1040	268.1040	0	Dh/SCG/HB/DS/DG
11	1.429	Guanosine	C_10_H_13_N_5_O_5_	[M-H] ^–^	282.0859	282.0844	-5.3	[M+H] ^+^	284.1001	284.0989	-4.2	DG
12	1.479	Succinic acid	C_4_H_6_O_4_	[M-H] ^–^	117.0193	117.0193	0	—	—	—	—	DS/SCG/DG
13	1.483	Guanine	C_5_H_5_N_5_O	—	—	—	—	[M+H] ^+^	152.0558	152.0567	5.9	DG
14	1.545	Galloyl glucose	C_13_H_16_O_10_	[M-H] ^–^	331.0684	331.0671	-3.9	—	—	—	—	DH
15	1.715	Leucine	C_6_H_13_NO_2_	[M-H] ^–^	130.0874	130.0874	0	[M+H] ^+^	132.1021	132.1019	-1.5	DG
16	1.794	Gallic acid	C_7_H_6_O_5_	[M-H] ^–^	169.0146	169.0142	-2.4	[M+H] ^+^	171.0288	171.0288	0	CS/DG
17	1.976	Gastrodine	C_13_H_18_O_7_	[M+HCOO] ^–^	331.1037	331.1035	-0.6	—	—	—	—	SCG
18	2.357	Gallic acid -O-diglucoside	C_19_H_26_O_15_	[M-H] ^–^	493.1211	493.1199	-2.4	—	—	—	—	DH
19	3.335	Woodorien	C_14_H_18_O_9_	[M-H] ^–^	329.0890	329.0878	-3.6	—	—	—	—	DS
20	3.621	9,10-dihydro-2-methyl-anthracen	C_15_H_14_	—	—	—	—	[M+H] ^+^	195.1169	195.1168	-0.5	HZ
21	3.870	Codonopsine	C_14_H_21_NO_4_	—	—	—	—	[M+H] ^+^	268.1503	268.1543	14.9	DS
22	4.130	Catechin 7-O-β-D-glucopyranoside	C_21_H_24_O_11_	[M-H] ^–^	451.1258	451.1246	-2.7	—	—	—	—	DH
23	6.383	Chlorogenic acid	C_16_H_18_O_9_	[M-H] ^–^	353.0891	353.0878	-3.7	—	—	—	—	DG/HB
24	5.080	Tryptophan	C_11_H_12_N_2_O_2_	[M-H] ^–^	203.0832	203.0826	-3	[M+H] ^+^	205.0952	205.0972	9.8	DS/DG
25	5.472	p-Hydroxybenzyl malonic acid	C_10_H_10_O_5_	[M-H] ^–^	209.0462	209.0455	-3.3	—	—	—	—	GC
26	5.472	Paeonol	C_9_H_10_O_3_	[M-H] ^–^	165.0561	165.0557	-2.4	—	—	—	—	CS
27	5.494	Emodin-O-(O-acetyl)-glucopyranoside	C_23_H_22_O_11_	—	—	—	—	[M+H] ^+^	475.1226	475.1235	1.9	DH
28	6.234	(-)-epicatechin	C_15_H_14_O_6_	[M-H] ^–^	289.0727	289.0718	-3.1	[M+H] ^+^	291.0850	291.0863	4.5	DH/TFL
29	4.478	Neochlorogenic acid	C_16_H_18_O_9_	[M-H] ^–^	353.0891	353.0878	-3.7	—	—	—	—	HB
30	6.649	digalloyl-glucoside	C_27_H_24_O_18_	[M-H] ^–^	635.0905	635.0890	-2.4	—	—	—	—	DH
31	6.654	Umbelliferone	C_9_H_6_O_3_	—	—	—	—	[M+H] ^+^	163.0381	163.0390	5.5	Dh
32	6.897	Cryptochlorogenin acid	C_16_H_18_O_9_	[M-H] ^–^	353.0891	353.0878	-3.7	[M+H] ^+^	355.1017	355.1024	2	HB
33	6.947	3-O-Feruloylquinic acid	C_17_H_20_O_9_	[M-H] ^–^	367.1046	367.1035	-3	—	—	—	—	HB
34	6.947	Berberrubine	C_19_H_16_NO_4_	[M+HCOO] ^–^	367.1052	367.1061	2.5	—	—	—	—	HB
35	7.046	Caffeic acid	C_9_H_8_O_4_	[M-H] ^–^	179.0357	179.0350	-3.9	[M+H] ^+^	181.0484	181.0495	6.1	DG
36	7.212	Vanillic acid	C_8_H_8_O_4_	[M-H] ^–^	167.0354	167.0350	-2.4	[M+H] ^+^	169.0490	169.0495	3	DG/DS/SCG
37	7.267	O-(O-methoxyphenoxy) phenol	C_13_H_12_O_3_	—	—	—	—	[M+H] ^+^	217.0871	217.0859	-5.5	DS
38	8.223	Isomer of liquiritin-O-glucoside	C_32_H_40_O_18_	[M-H] ^–^	711.2143	711.2142	-0.1	—	—	—	—	GC
39	8.510	(+)-Catechin	C_15_H_14_O_6_	[M-H] ^–^	289.0727	289.0718	-3.1	[M+H] ^+^	291.0850	291.0859	4.5	DG/DH/TFL
40	8.643	(p-Hydroxybenzul)-6,7-dihydroxy-	C_23_H_29_NO_8_	—	—	—	—	[M+H] ^+^	448.1957	448.1966	2	HB
		N-metylterahydroisoquinoline-7-O-										
		β-D-glucopyranoside										
41	8.659	7-O-Glucoptranoside of 1-(p-hydroxy	C_23_H_30_NO_8_	—	—	—	—	[M^+^H]^+^	449.2013	449.2044	6.9	HB
		benzyl)-6,7-dihydroxy-N-methylisoquinoline										
42	8.703	Kaempferol-3-O-rutinose	C_27_H_30_O_15_	[M-H] ^–^	593.1541	593.1512	-4.9	—	—	—	—	DG
43	8.803	Phellodendrine	C_20_H_24_NO_4_	[M+CI] ^–^	376.1331	376.1321	-2.7	—	—	—	—	HB
44	9.035	Hexyl-β-D-glucoside sulfate	C_12_H_24_O_9_S	[M+CH_3_COO] ^–^	403.1250	403.1280	7.4	—	—	—	—	DS
45	9.057	Apigenin-6,8-di-C-β-D-glucopyranoside	C_27_H_30_O_15_	—	—	—	—	[M+H]^+^	595.1645	595.1657	2	GC
46	9.140	Tetrehydropalmatrubine	C_20_H_23_NO_4_	[M-H] ^–^	340.1497	340.1554	16.8	[M+H]^+^	342.1690	342.1700	2.9	HB
47	9.233	Amurenlaetone B	C_17_H_20_O_9_	[M-H] ^–^	367.1045	367.1035	-2.7	—	—	—	—	HB
48	9.538	1-O-galloyl-2-O-cinnamoyl-β-D-glucose	C_22_H_22_O_11_	—	—	—	—	[M+H] ^+^	463.1225	463.1235	2.2	DH
49	9.797	Albiflorin	C_23_H_28_O_11_	[M+HCOO] ^–^	525.1634	525.1614	-3.8	—	—	—	—	CS/DG
50	9.797	Benzoylpaeoniflorin	C_24_H_24_O_10_	[M+HCOO] ^–^	517.1324	517.1351	5.2	—	—	—	—	DG
51	10.151	4-Hydroxy-3-methyl-acetophenone	C_9_H_10_O_2_	—	—	—	—	[M+H] ^+^	151.0740	151.0754	9.3	CS/Dh
52	10.350	Isoliensinine	C_19_H_23_NO_3_	[M-H] ^–^	312.1613	312.1605	-2.6	[M+H] ^+^	314.1743	314.1751	2.5	HB
53	10.592	Galloylpaeoniflorin	C_34_H_28_O_22_	[M-H] ^–^	787.1008	787.0999	-1.1	—	—	—	—	CS
54	10.625	Cyclohexanecarboxylic acid	C_17_H_20_O_9_	—	—	—	—	[M+H] ^+^	369.1165	369.1180	4.1	HB
55	10.830	Astragaloside V/VI/VII	C_26_H_28_O_13_	—	—	—	—	[M+H] ^+^	549.1595	549.1603	1.5	DG
56 *^b^*	10.973	Ferulic acid	C_10_H_10_O_4_	[M-H] ^–^	193.0510	193.0506	-2.1	[M+H] ^+^	195.0644	195.0652	4.1	DG/Dh/HB/DS
57 *^b^*	10.975	Polydatin	C_20_H_22_O_8_	[M-H] ^–^	389.1258	389.1242	-4.1	—	—	—	—	HZ
55	10.830	Astragaloside V/VI/VII	C_26_H_28_O_13_	—	—	—	—	[M+H] ^+^	549.1595	549.1603	1.5	DG
56 *^b^*	10.973	Ferulic acid	C_10_H_10_O_4_	[M-H] ^–^	193.0510	193.0506	-2.1	[M+H] ^+^	195.0644	195.0652	4.1	DG/Dh/HB/DS
57 *^b^*	10.975	Polydatin	C_20_H_22_O_8_	[M-H] ^–^	389.1258	389.1242	-4.1	—	—	—	—	HZ
58	11.029	Atractylenolide III	C_15_H_20_O_3_	—	—	—	—	[M+H] ^+^	249.1469	249.1485	6.4	CZ/DS
59	11.404	Raffinose	C_18_H_32_O_16_	[M+HCOO] ^–^	549.1632	549.1672	7.3	—	—	—	—	DS
60 *^b^*	11.470	Astilbin	C_21_H_22_O_11_	[M-H] ^–^	449.1109	449.1089	-4.5	[M+H] ^+^	451.1228	451.1235	1.6	TFL
61	11.570	Cinnamic acid	C_9_H_8_O_2_	[M-H] ^–^	147.0450	147.0452	1.4	[M+H] ^+^	149.0588	149.0597	6	SCG
62	11.725	Liquiritigenin	C_15_H_12_O_4_	—	—	—	—	[M+H] ^+^	257.0800	257.0808	3.1	GC
63	11.818	(+/-)-8-(4-Hydroxy-3-methoxyphenyl)-	C_27_H_36_O_13_	[M-H] ^–^	567.2095	567.2083	-2.1	—	—	—	—	HB
		6,7-bis(hydroxymethyl)-3-methoxy-5,6,										
		7,8-tetrahydro-2-naphthalenyl-β-D-										
		glucopyranoside										
64	12.017	Ferulic acid-1-O-glucoside	C_16_H_20_O_9_	[M-H] ^–^	355.1047	355.1035	-3.4	—	—	—	—	DH
65	12.166	Galloylpaeoniflorinb	C_30_H_32_O_15_	[M-H] ^–^	631.1678	631.1668	-1.6	—	—	—	—	DG
66	12.289	Taxifolin	C_15_H_12_O_7_	—	—	—	—	[M+H] ^+^	305.0645	305.0656	3.6	TFL
67	12.769	Galloyl cinnamoyl-glucose	C_22_H_22_O_11_	—	—	—	—	[M+H] ^+^	463.1229	463.1235	1.3	DH
68	13.078	Quercitrin	C_21_H_20_O_11_	[M-H] ^–^	447.0944	447.0933	-2.5	—	—	—	—	TFL
69	13.084	Oxidized jatrorrhizine	C_20_H_17_NO_5_	—	—	—	—	[M+H] ^+^	352.1169	352.1179	2.8	HB
70	13.160	Laccaic acid D-O-glucose	C_22_H_20_O_12_	[M-H] ^–^	475.0897	475.0882	-3.2	—	—	—	—	DH
71	13.283	Isomer of liquiritin apioside	C_25_H_24_O_11_	—	—	—	—	[M+H] ^+^	501.1400	501.1391	-1.8	GC
72	13.310	Engeletin	C_21_H_22_O_10_	[M-H] ^–^	433.1152	433.1140	-2.8	—	—	—	—	TFL
73	13.343	Procyanidine/isomer	C_16_H_20_O_9_	[M-H] ^–^	355.1047	355.1035	-3.4	—	—	—	—	DH
74	13.359	Resveratrol-4-O-β-D-(2′-O-galloyl)-	C_27_H_26_O_12_	[M-H] ^–^	541.1367	541.1351	-3	—	—	—	—	DH
		glucopyranoside or resveratrol-4-O-β-										
		D-(6′-O-galloyl)-glucopyranoside										
75	13.558	Dactylorhin A	C_40_H_56_O_22_	[M-H] ^–^	887.3196	887.3190	-0.7	—	—	—	—	SCG
76	13.581	Columbianetin	C_14_H_14_O_4_	—	—	—	—	[M+H] ^+^	247.0956	247.0965	3.6	Dh
77	13.790	Azelaic acid	C_9_H_16_O_4_	[M-H] ^–^	187.0982	187.0976	-3.2	—	—	—	—	DS
78	13.989	(6aR, 11aR)-3-hydroxy-9, 10-dimethoxy-	C_25_H_24_O_12_	[M-H] ^–^	515.1209	515.1195	-2.7	—	—	—	—	DG
		pterocarpan-3-O-β-D-glc-6′′-O-malonate										
79 *^b^*	14.394	Paeoniflorin	C_23_H_28_O_11_	—	—	—	—	[M+H] ^+^	481.1570	481.1704	27.8	CS
80	14.510	3-methoxy-4-hydroxyphenylethanol	C_9_H_12_O_3_	—	—	—	—	[M+Na] ^+^	191.0692	191.0679	-6.8	SCG
81 *^b^*	14.718	Atractylodin	C_13_H_10_O	[M+HCOO] ^–^	227.0721	227.0714	-3.1	—	—	—	—	CZ
82	14.990	Aloe-emodin-8-O-β-D-glucopyranoside	C_21_H_20_O_10_	—	—	—	—	[M+Na] ^+^	455.0948	455.0949	0.2	DH
83	15.182	1-O-galloyl-2-O-cinnamoyl-β-D-glucose	C_22_H_22_O_11_	[M-H] ^–^	461.1105	461.1089	-3.5	—	—	—	—	DH
84 *^b^*	15.265	Liquiritin	C_21_H_22_O_9_	[M-H] ^–^	417.1157	417.1191	8.2	[M+H] ^+^	419.1332	419.1337	1.2	GC
85	15.281	Acetyl-liquiritin/isoliquiritin	C_23_H_24_O_10_	[M-H] ^–^	459.1306	459.1297	-2	—	—	—	—	GC
86	15.935	Ononin	C_22_H_22_O_9_	—	—	—	—	[M+H] ^+^	431.1306	431.1337	7.2	DG/GC
87	16.160	Licorice glycoside B	C_35_H_36_O_15_	[M-H] ^–^	695.1989	695.1981	-1.2	—				GC
88	16.482	Hexyl-b-D-glucopyranosyl-(1/6)-β-D-	C_18_H_34_O_11_	—	—	—	—	[M+H] ^+^	427.2146	427.2174	6.8	DS
		glucopyranoside										
89	16.524	Militarine	C_34_H_46_O_17_	[M-H] ^–^	725.2670	725.26662	-1.1	—	—	—	—	SCG
90	16.988	Cinnamic-O-galloyl-glucoside	C_22_H_22_O_11_	[M-H] ^–^	461.1104	461.1089	-3.3	—	—	—	—	DH
91	18.181	Isoengeletin	C_21_H_21_O_10_	[M-H] ^–^	432.1029	432.1062	7.6	—	—	—	—	TFL
92	18.222	Senkyunolide H	C_12_H_16_O_4_	—	—	—	—	[M+Na] ^+^	247.0958	247.0941	-6.9	DG
93 *^b^*	18.413	Chrysophanol	C_15_H_10_O_4_	[M-H] ^–^	253.0509	253.0506	-1.2	[M+H] ^+^	255.0642	255.0652	3.9	DH/SCG
94 *^b^*	18.520	Aloe-emodin	C_15_H_10_O_5_	—	—	—	—	[M+H] ^+^	271.0596	271.0601	1.8	DH
95	19.291	22-Acetoxyl licorice-saponin G2	C_44_H_64_O_19_	[M-H] ^–^	895.3978	895.3969	-1	—	—	—	—	GC
96	19.556	22-Hydroxy-licorice-saponin G2	C_42_H_62_O_18_	[M-H] ^–^	853.3871	853.3863	-0.9	—	—	—	—	GC
97	20.269	Licorice-saponin A3 (or isomer)	C_48_H_72_O_21_	[M-H] ^–^	983.4492	983.4493	0.1	—	—	—	—	GC
98	20.551	Calycosin-7-O-β-D-glucoside	C_22_H_22_O_10_	[M-H] ^–^	445.1148	445.1140	-1.8	—	—	—	—	DG/GC
99	20.608	N-Methylcanadine	C_21_H_24_NO_4_	—	—	—	—	[M+Na] ^+^	377.1591	377.1598	1.9	HB
100 *^b^*	20.857	Physcion	C_16_H_12_O_5_	[M-H] ^–^	283.0619	283.0612	-2.5	[M+H] ^+^	285.0751	285.0757	2.1	DH/SCG
101	20.865	22β-Acetoxyl-glycyrrhizin	C_44_H_64_O_18_	[M-H] ^–^	879.4027	879.4020	-0.8	—	—	—	—	GC
102	21.130	Uralsaponin E	C_42_H_60_O_17_	[M-H] ^–^	835.3799	835.3758	-4.9	—	—	—	—	GC
103	21.313	Uralsaponin U/Uralsaponin N	C_42_H_62_O_17_	[M-H] ^–^	837.3922	837.3914	-1	—	—	—	—	GC
104	21.470	(6a*R*, 11a*R*)-3-Hydroxy-9, 10-	C_17_H_16_O_5_	—	—	—	—	[M+H] ^+^	301.1070	301.1071	0.3	DG
		dimethoxypterocarpan										
105	21.719	Phellodenol D	C_17_H_18_O_5_	—	—	—	—	[M+H] ^+^	303.1230	303.1227	-1	HB
106	21.876	Shancigusin H	C_49_H_62_O_23_	[M-H] ^–^	1017.3613	1017.3609	-0.4	—	—	—	—	SCG
107	22.307	Laccaic acid D-8-O-(6′-O-cinnamoyl)-	C_31_H_26_O_13_	[M-H] ^–^	605.1307	605.1301	-1	—	—	—	—	DH
		glucopyranoside										
108	22.539	(6R, 9S)-3-Oxo-α-ionol-β-D-	C_19_H_30_O_10_S	[M-H] ^–^	449.1469	449.1487	4	—	—	—	—	DS
		glucopyranoside sulfate										
109	22.597	Bicyclo [3.1.0] hexan-3-one	C_12_H_18_O_2_	—	—	—	—	[M+H] ^+^	195.1366	195.1380	7.2	Dh
110	22.920	Licorice-saponin G2	C_42_H_62_O_17_	[M-H] ^–^	837.3932	837.3914	−2.1	—	—	—	—	GC
111	23.244	(+) N-methylcorydine	C_21_H_26_NO_4_	—	—	—	—	[M+Na] ^+^	379.1740	379.1754	3.7	HB
112	24.014	22*β*-Acetoxy-licorice-saponin B2	C_44_H_66_O_17_	[M-H] ^–^	865.4229	865.4227	−0.2	—	—	—	—	GC
113	24.279	Macedonoside C	C_42_H_62_O_16_	[M-H] ^–^	821.3972	821.3965	−0.9	—	—	—	—	GC
114	24.487	Obaculactone	C_26_H_30_O_8_	—	—	—	—	[M+H] ^+^	471.2009	471.2013	0.8	HB
115 *^b^*	24.676	Rhein	C_15_H_8_O_6_	[M-H] ^–^	283.0258	283.0248	−3.5	[M+H] ^+^	285.0390	285.0394	1.4	DH
116	24.702	Glycyrrhetic acid	C_30_H_46_O_4_	—	—	—	—	[M+H] ^+^	471.3467	471.3469	0.4	GC
117	25.588	Glycyrflavoside C	C_42_H_64_O_15_	[M-H] ^–^	807.4180	807.4172	−1	—	—	—	—	GC
118	25.614	trans-Resveratrol	C_14_H_12_O_3_	—	—	—	—	[M+H] ^+^	229.0857	229.0859	0.9	TFL/HZ
119 *^b^*	25.869	Glycyrrhizic acid	C_42_H_62_O_16_	[M-H] ^–^	821.3975	821.3965	−1.2	—	—	—	—	GC
120	27.652	Phytosphingosine	C_18_H_39_NO_3_	—	—	—	—	[M+H] ^+^	318.2991	318.3003	3.8	DG
121	28.829	Octanal	C_8_H_16_O	—	—	—	—	[M+H] ^+^	129.1266	129.1274	6.2	DS/Dh
122 *^b^*	29.448	Emodin	C_15_H_10_O_5_	[M-H] ^–^	269.0465	269.0455	−3.7	[M+H] ^+^	271.0596	271.0601	1.8	DS/DH/SCG/HZ
123	29.448	Z-11-hexadecenoic acid	C_16_H_30_O_2_	[M+CH_3_COO] ^–^	313.2393	313.2384	−2.9	—	—	—	—	Dh
124	29.458	2, 6-dimethoxy-4-(2’-propenyl) phenol	C_11_H_14_O_3_	—	—	—	—	[M+H] ^+^	195.1016	195.1016	0	DS
125	29.889	Ligustilide	C_12_H_14_O_2_	—	—	—	—	[M+H] ^+^	191.1063	191.1067	2.1	DG
126 *^b^*	30.254	Osthole	C_15_H_16_O_3_	—	—	—	—	[M+H] ^+^	245.1173	245.1172	−0.4	DG/Dh
127 *^b^*	31.116	Columbianadin	C_19_H_20_O_5_	—	—	—	—	[M+H] ^+^	329.1387	329.1384	−0.9	Dh
128	31.271	9-Tetradecenoic acid	C_14_H_26_O_2_	[M+CH_3_COO] ^–^	285.2081	285.2071	−3.5	—	—	—	—	Dh
129	31.652	(E)-2-Hexenyl-β-D-glucopyranosyl-	C_18_H_32_O_11_	[M-H] ^–^	423.1860	423.1872	2.8	—	—	—	—	DS
		(1/2)-β-D-glucopyranoside										
130	31.917	α-Selinene	C_14_H_20_	[M+HCOO] ^–^	233.1554	233.1547	−3	—	—	—	—	CZ
131	33.353	Dibutylphthalat	C_16_H_22_O_4_	—	—	—	—	[M+H] ^+^	279.1598	279.1591	−2.5	HZ
132	34.563	Citronellyl acetate	C_12_H_22_O_2_	—	—	—	—	[M+H] ^+^	199.1698	199.1693	−2.5	CZ
133	34.646	Litcubine	C_19_H_22_NO_4_	—	—	—	—	[M+H] ^+^	329.1605	329.1622	5.2	HB
134 *^b^*	34.834	Oleanolic acid	C_30_H_48_O_3_	[M-H] ^–^	455.3532	455.3531	−0.2	—	—	—	—	WLX
135	35.546	Tetradecanoic acid	C_14_H_28_O_2_	[M-H] ^–^	227.2022	227.2017	−2.2	—	—	—	—	DS
136	35.624	1,2-Benzenedicarboxylic acid	C_24_H_38_O_4_	—	—	—	—	[M+H] ^+^	391.2852	391.2843	−2.3	DS
		diisooctyl ester										
137	36.192	Linoleate	C_18_H_32_O_2_	[M-H] ^–^	279.2340	279.2330	−3.6	—	—	—	—	DG
138	36.325	Octadecanoic acid	C_18_H_36_O_2_	[M-H] ^–^	283.2650	283.2643	−2.5	—	—	—	—	DG
139	37.170	Hexadecanoic acid	C_16_H_32_O_2_	[M-H] ^–^	255.2340	255.2330	−3.9	—	—	—	—	DS/DG/Dh/SCG

^a^ TFL, Smilacis Glabrae Rhizome; CS, Paeoniae Radix Rubra; HZ, Polygoni Cuspidati Rhizoma et Radix; SCG, Cremastrae Pseudobulbus; DH, Rhei Radix et Rhizoma; WLX, Clematidis Radix et Rhizome; Dh, Angelicae Pubescentis Radix; HB, Phellodendri Chinensis Cortex;CZ, Atractylodis Rhizome; GC, Glycyrrhizae Radix et Rhizome; DS, Codonopsis Radix; DG, Angelicae Sinensis Radix.

^b^ Components identified with reference compounds comparison.

Among them, 15 compounds (compounds **56**, **57**, **60**, **79**, **81**, **84**, **93**, **94**, **100**, **115**, **119**, **122**, **126**, **127,** and **134**) were identified as ferulic acid, polydatin, astilbin, paeoniflorin, atractylodin, liquiritin, chysophanol, aloe-emodin, physcion, rhein, glycyrrhizic acid, emodin, osthole, columbianadin, and oleanolic acid by comparing the retention time, quasi-molecular ions with authentic standards, respectively. While the others were tentatively deduced based on their high-accurate quasi-molecular ion such as [M−H]^−^, [M+HCOO]^−^, [M+CH_3_COO]^−^, [M]^+^, [M+H]^+^, and [M+Na]^+^ with those of the known published compounds recorded in the in-house library. Information regarding the 139 constituents, such as t_R_ (min), identification, formula, negative ion (m/z), positive ion (m/z), and botanical source, is offered in **Table 1**.

### Network Pharmacology Analysis

#### Gouty Arthritis-Related Targets Network Analysis

**Figure 3:**
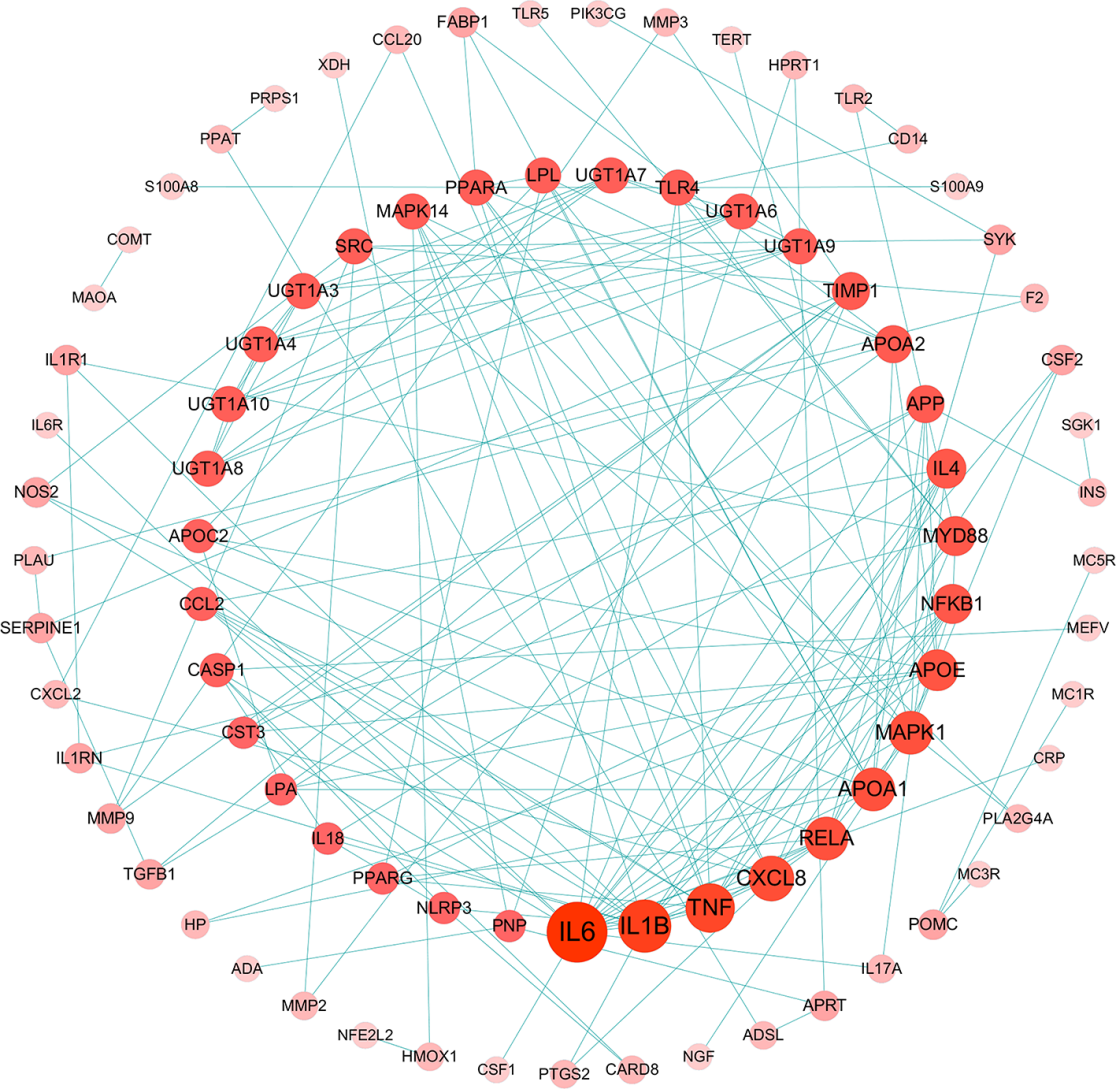
Gouty arthritis-related targets of protein-protein interaction (PPI) network (Confidence score >0.95, Node size represent the degree).

The relationship among 175 disease genes from PPI were subjected to STRING. And a gene-gene interaction network was accordingly achieved. Then, disease targets of PPI with high confidence score (>0.95) were screened for network construction and analysis (**Figure 3**), 84 nodes and 171 edges were involved in this network. Among them, the central part notes connected by more than 4 edges, such as 19 in IL-6, 15 in IL-1β, 5 in CASP1, and 4 in NLRP3. It implies that these genes might be key nodes in this network.

#### Potential Target Genes and Network Construction

**Figure 4:**
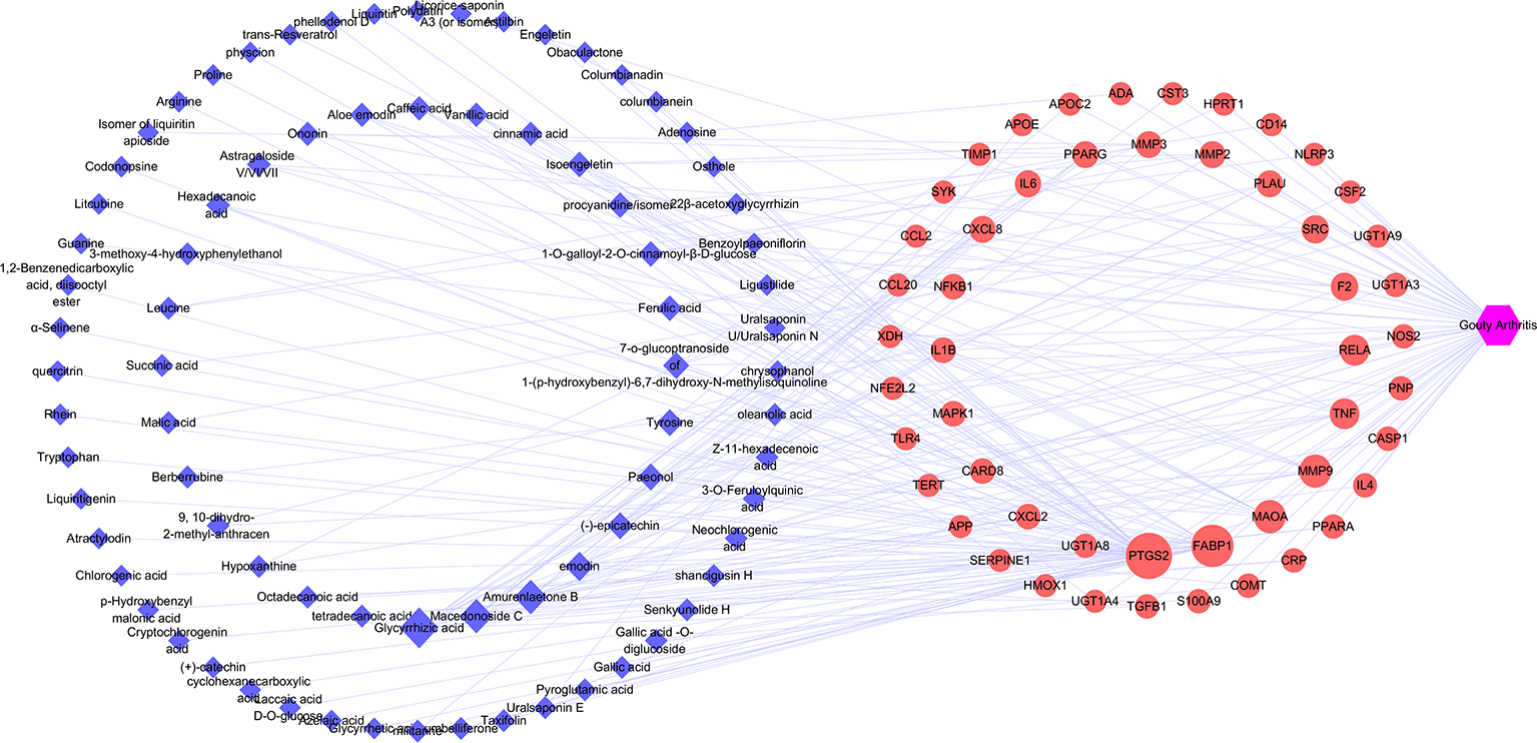
Compound-potential targets-disease network (Blue diamond represents compound target; red circle represents pathway; purple hexagon represents disease).

The target genes related to gouty arthritis and compounds were got from associated databases, and 51 overlapping genes were identified by matching the aforementioned genes. Then compound-potential targets-disease network constructed by Cytoscape was shown in **Figure 4**, which comprise of 132 nodes (1 disease node, 51 potential target nodes, and 80 compound nodes) and 236 edges. From this network, we can conclude that glycyrrhizic acid, amurenlaetone B, and macedonoside C may be the main components of JSCBR in treating gouty arthritis due to higher degree.

#### Potential Target Gene-Related Pathway Analysis

**Figure 5:**
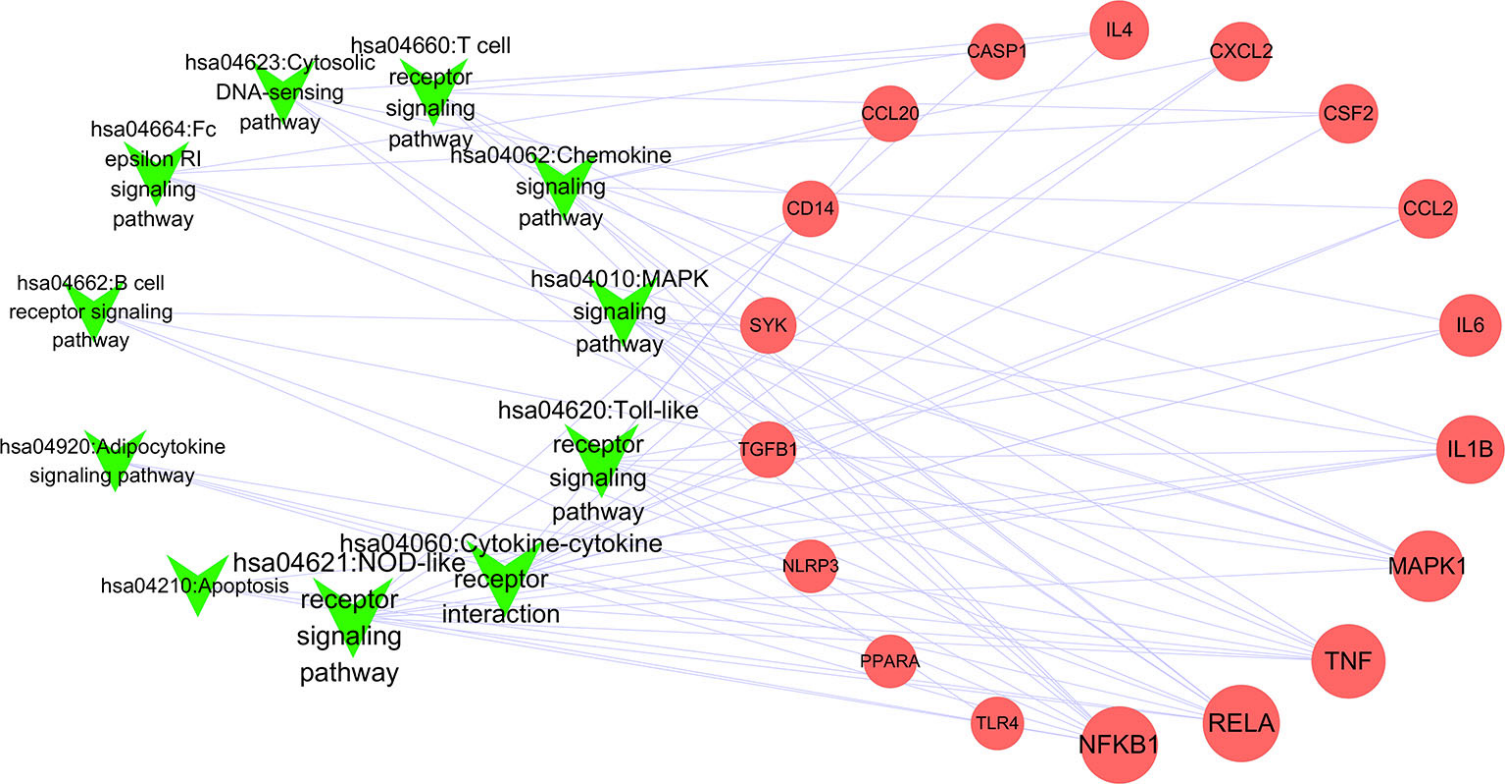
Network of pathways and related genes (Green “V” represents pathways; red circle represents pathway; node size represents the degree).

To better understand the signaling pathways and functions of these potential target genes, we performed functional enrichment analysis using KEGG pathways. The identified pathways and genes in which they are involved are shown in **Figure 5** and **Table 2** (p < 0.05). NOD-like receptor signaling pathway (hsa04621) is ranked first, which has 10 genes involved. In our present research, NRLP3/ASC/CASP1/IL1B was selected for experimental verification.

**Table 2:** Functions of potential target genes based on KEGG pathway analysis.

No	Term	P Value	Genes
1	hsa04621: NOD-like receptor signaling pathway	1.24E-11	MAPK1, CARD8, IL6, TNF, CCL2, RELA, CXCL2, IL1B, NFKB1, CASP1, NLRP3
2	hsa04620: Toll-like receptor signaling pathway	7.54E-06	MAPK1, IL6, TNF, RELA, IL1B, NFKB1, TLR4, CD14
3	hsa04060: Cytokine-cytokine receptor interaction	5.65E-04	IL4, CSF2, IL6, TNF, CCL2, CCL20, CXCL2, IL1B, TGFB1
4	hsa04623: Cytosolic DNA-sensing pathway	6.88E-04	IL6, RELA, IL1B, NFKB1, CASP1
5	hsa04660: T cell receptor signaling pathway	0.0011312	IL4, CSF2, MAPK1, TNF, RELA, NFKB1
6	hsa04664: Fc epsilon RI signaling pathway	0.0025462	IL4, CSF2, MAPK1, TNF, SYK
7	hsa04062: Chemokine signaling pathway	0.011938	MAPK1, CCL2, CCL20, RELA, CXCL2, NFKB1
8	hsa04010: MAPK signaling pathway	0.0132372	MAPK1, TNF, RELA, IL1B, NFKB1, CD14, TGFB1
9	hsa04920: Adipocytokine signaling pathway	0.0132749	PPARA, TNF, RELA, NFKB1
10	hsa04662: B cell receptor signaling pathway	0.0179629	MAPK1, RELA, NFKB1, SYK
11	hsa04210: Apoptosis	0.0265251	TNF, RELA, IL1B, NFKB1

### Experimental Validation Using Molecular Cell Biology

#### Effect of JSCBR Extracts on Cell Viability

**Figure 6:**
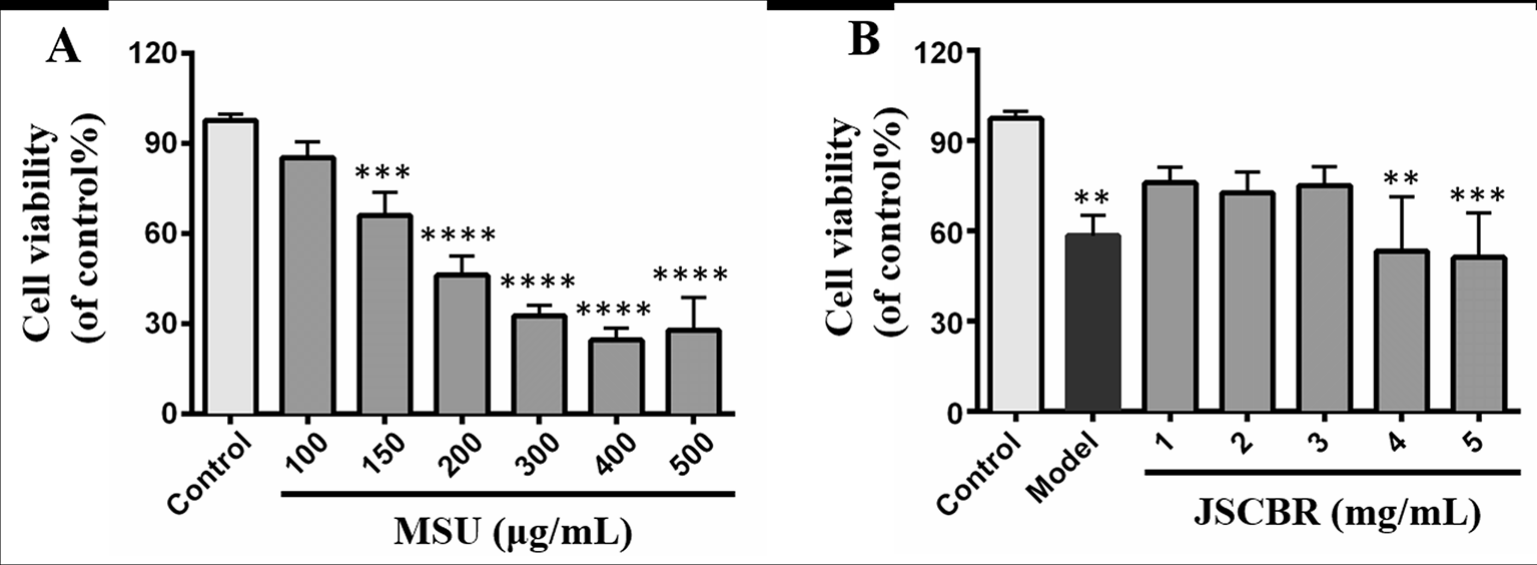
Effects of Jiang-Suan-Chu-Bi recipe (JSCBR) extracts on monosodium urate (MSU)-induced THP-1 cell viability. **(A)** THP-1 cells were exposed to MSU at various concentrations for 24 h. **(B)** Protective effects of JSCBR extracts on the viabilities of MSU-induced THP-1 cells. Cell viability was assessed by CCK-8 assay and expressed relative to untreated control cells. **p < 0.01, ***p < 0.001, ****p < 0.0001 versus control group.

The induction effect of MSU was evaluated *in vitro* with a CCK-8 assay and shown in **Figure 6A**. Notably, MSU acted as the strongest inducer decreased the viabilities of THP-1 cell in a concentration-dependent manner. Since the relatively low viability was observed in cells exposed to MSU with dosages greater than or equal to 200 μg/ml, 150 μg/ml was the optimum induction dosage in further experiments. For antiinflammatory activity, THP-1 cells treated with JSCBR extracts from 1 to 5 mg/ml exhibited viability of 67.8%~80.6% (**Figure 6B**). Since no further antiproliferation effect was observed in cells exposed to 4 and 5 mg/ml, the concentrations of extracs were defined to 1, 2, and 3 mg/ml for western Blot verification.

### Western Blot Analysis

**Figure 7:**
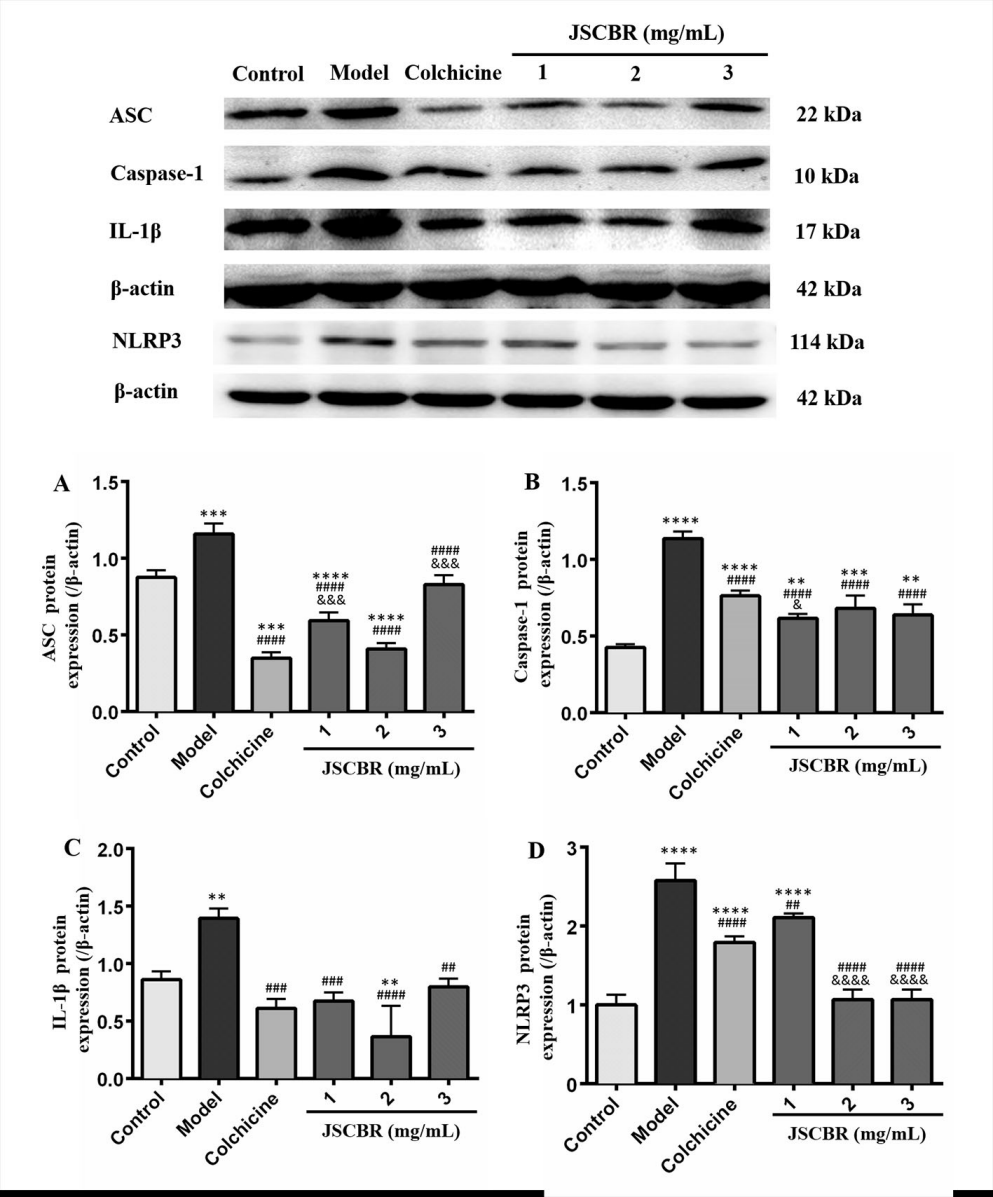
Jiang-Suan-Chu-Bi recipe (JSCBR) extracts protect THP-1 cells against monosodium urate (MSU)-induced inflammation by affecting the expression of proteins from the NOD-like receptor signaling pathway. **(A)** Effects of JSCBR extracts on ASC, caspase-1, IL-1β, and NLRP3 protein levels in MSU-induced THP-1 cells based on the western blotting assay; **(B)** Statistical analysis of the effects of JSCBR extracts on protein expressions levels. Data are presented as the mean ± SD (*n* = 3), **p < 0.01, ***p < 0.001, ****p < 0.0001 versus control group. ^##^ *p* < 0.01, ^###^ *p* < 0.001, ^####^ *p* < 0.0001 versus model group. ^&^ *p* < 0.05, ^&&&^ *p* < 0.001, ^&&&&^ *p* < 0.0001 versus colchicine group.

In order to validate the action mechanism of JSCBR screened out by phytochemistry-based network pharmacology, protein expression of ASC, caspase-1, IL-1β, and NLRP3 was examined by Western Blot Analysis. Compared with a control group, the expression of these three proteins in the model group was significantly increased (**Figure 7**, P < 0.01), while these protein expression changes were attenuated by treatment with colchicine and different concentrations of JSCBR extracts (1, 2, and 3 mg/ml) (**Figure 7**, P < 0.01). The results suggested that the antiinflammation of JSCBR on gouty arthriris was associated with inhibition of ASC, caspase-1, IL-1β, and NLRP3 protein expression, which belongs to NOD-like receptor signaling pathway.

## Discussion

In recent years, prevalence of gouty arthritis annually increased with the continuous improvement of people’s living standards. Although some achievement has been made in reducing the mortality of the disease, it still imposed a huge economic burden on patients and society which also reduced the quality of life of patients. Colchicine, glucocorticoids, and nonsteroidal antiinflammatory drugs, acted as the current mainstay drugs for gouty arthritis, have been controversial due to their various side effects. It is very necessary to develop new drugs with remarkable curative effect and little side effect.

The pathogenesis of gouty arthritis is classified in the “damp-heat” category according to TCM theory, the therapeutic principles of “clearing heat, dispelling dampness, dissipating stasis, and relieving pain” should be used. JSCBR, a formula prescribed according to abovementioned principles and utilization frequency and made up of 12 kinds of herbs, which has been effective in treating gouty arthritis. However, its “multicomponents”, “multitargets” and “multipathways” features make it much difficult to decipher the molecular mechanisms of JSCBR in the treatment of gouty arthritis from a systematic perspective. In this study, we combined phytochemistry, network pharmacology and molecular cell biology to evaluate the effective components and possible molecular mechanisms of JSCBR in the treatment of gouty arthritis (Dong et al., 2019).

Firstly, chemical profile of JSCBR was characterized for the first time, which lays the material foundation for the follow-up network pharmacology research. Network pharmacological analysis of JSCBR identified 51 potential target nodes, 80 compounds, and 11 related pathways related to gouty arthritis. Among the target compounds linked to the network, two triterpenoid saponins glycyrrhizic acid (Compound **119**) from Glycyrrhizae Radix et Rhizome and macedonoside C (Compound **113**) from Rhei Radix et Rhizoma, as well as a phenolic acid amurenlaetone B (Compound **47**) from Phellodendri Chinensis Cortex may be the effective components of JSCBR in treating gouty arthritis due to higher degree. The two triterpenoid saponins could act at least in part, as a glucocorticoid-like drug due to the similar chemical structure related to glucocorticoids. Additionally, reports have provided evidence that treatments with these two types of components can alleviate the symptoms of gouty arthritis by exerting antiinflammatory and antioxidant pharmacological effects (Ling and Bochu, 2014; Zhou et al, 2017; Jhang et al, 2018).

Among the 51 putative targets and 11 related pathways of JSCBR associate with gouty arthritis, it was discovered that IL-6, IL-1β, CASP1, and NLRP3 were relatively important targets evaluated by topological parameters, which are involved in NOD-like receptor, Toll-like receptor, cytokine-cytokine receptor interaction, and cytosolic DNA-sensing signaling pathways. Among which, NOD-like receptor signaling pathways was ranked first. Thus, a further confirmation was selected for further verification.

**Figure 8:**
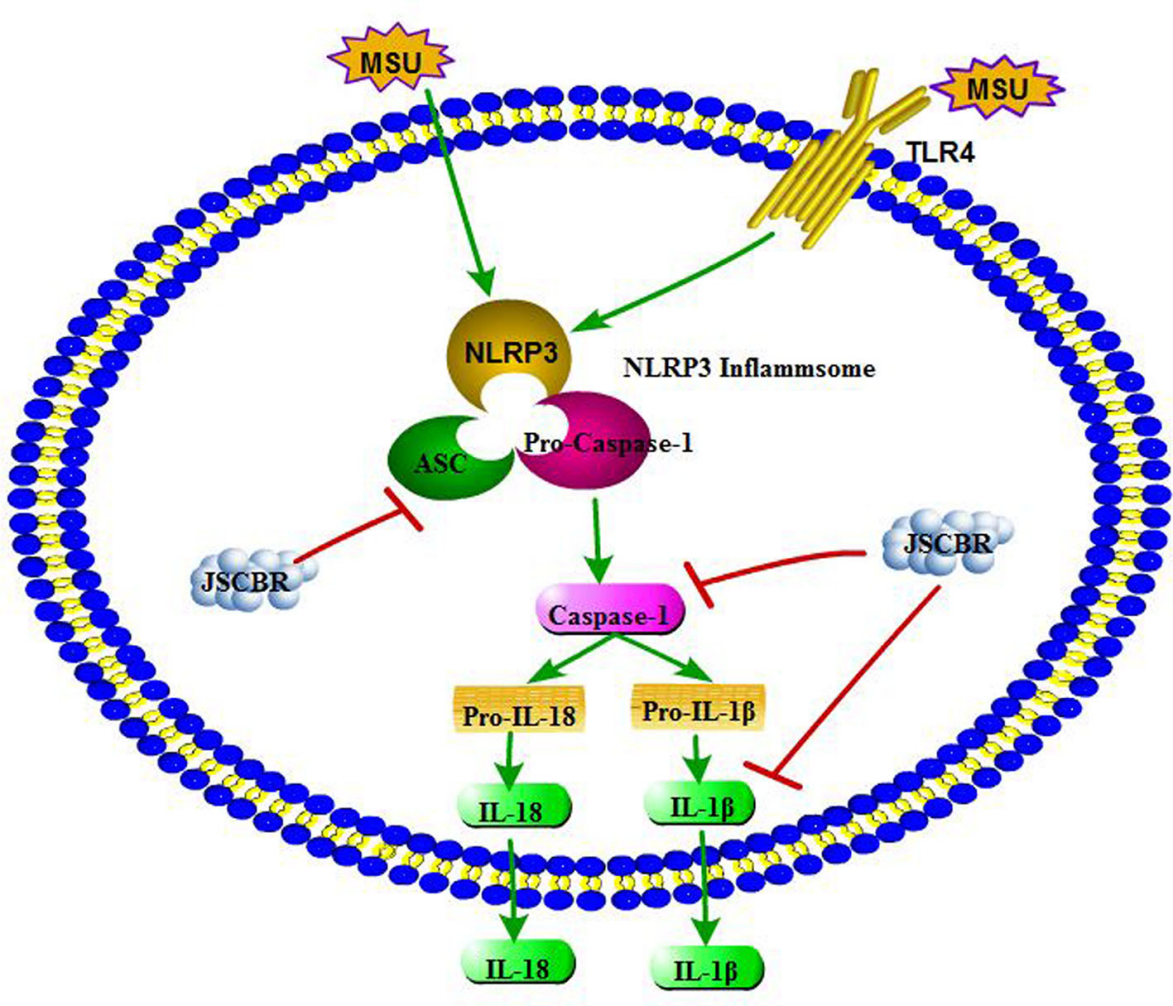
Overview of potential mechanisms underlying the protective effects of Jiang-Suan-Chu-Bi recipe (JSCBR) on monosodium urate (MSU)-induced gouty arthritis.

As illustrated in some research studies, NOD-like receptor protein-3 inflammasome (NLRP3) and toll-like receptor-4 (TLR4) plays an important role during gouty arthritis (**Figure 8**). NLRP3 inflammasome, an assembly composed of NLRP3, apoptosis-associated speck-like protein (ASC) containing a C-terminal caspase recruitment domain (CARD) and the effector procaspase-1, which can be activated by a “danger signal” crystal MSU. Once activated, NLRP3 oligomerizes and recruits the ASC adaptor protein and procaspase-1 sequentially. Caspase-1 was then activated and proteolytic cleavaged by the complex, resulting in specialized maturation and secretion of IL-1β and IL-18. In addition, MSU could also be recognized by Toll-like receptors, Toll-like receptor pathway could active the NLRP3 inflammasome complex and further induce an activation of inflammatory cascade (Mccormack et al., 2009; Xiao et al, 2015).

Our experiments in molecular cell biology had shown that the expressions of ASC, caspase-1, and IL-1β were significantly increased in the MSU-induced THP-1 cells, while they were significantly downregulated after treatment with JSCBR, which is consistent with network prediction. Therefore, it is speculated that NOD-like receptor signaling pathway is involved in the pathogenesis of gouty arthritis, and JSCBR could treat gouty arthritis by negative regulation of the inflammatory response. Besides, other potential mechanisms of JSCBR explored by above integrated strategy need for further investigation.

## Conclusion

In the current study, an integrated approach based on chemical profile, network pharmacology and experimental support using molecular cell biology were carried out to reveal the therapeutic composition and mechanisms of JSCBR against gouty arthritis. Following identification of 139 chemical constituents in JSCBR by UHPLC-QTOF MS, 175 disease genes, 51 potential target nodes, 80 compounds, and 11 related pathways were achieved by network pharmacology analysis. Potential key targets NRLP3/ASC/CASP1/IL1B that associated with the negative regulation of in NOD-like receptor signaling pathway were further verified in monosodium urate-induced THP-1 cells, and a plausible mechanism for the multitarget effects of JSCBR on gouty arthritis was consequently proposed (**Figure 8**). Taken together, our results provide a comprehensive insight into therapeutic composition and mechanistic of JSCBR, which make beneficial exploration for the research and development of traditional Chinese herbal formula.

## Data Availability Statement

All datasets generated for this study are included in the article/**Supplementary Material**.

## Author Contributions

NX and JQ wrote the manuscript and analyze data. NX, SH, PH, YQ conducted the research and developed the network pharmacology experiment. GL and TP revised the manuscript and edited the graphs. HS did the English proofreading of the manuscript. LZ designed the experiments and edited the manuscript. All the authors read and approved the final version of the manuscript.

## Funding

This work was financially supported by the National Natural Science Foundation of China (No. 81873195 and 81703675), Liaoning Natural Science Foundation (20180550980 and 20180550593), Liaoning Revitalization Talents Program (XLYC1907113), 2018 program for Liaoning Excellent Talents in University, the project of Dalian Young Star on Science and Technology in 2016 (2017RQ122) and Dalian Municipal Medical Research Foundation (no. 17Z2001).

## Conflict of Interest

The authors declare that the research was conducted in the absence of any commercial or financial relationships that could be construed as a potential conflict of interest.

## Supplementary Material

Table S1 Therapeutic targets of gouty arthritis.

Table S2 Compound targets for JSCBR.
